# Heat Shock Proteins 70 Regulate Cell Motility and Invadopodia-Associated Proteins Expression in Oral Squamous Cell Carcinoma

**DOI:** 10.3389/fendo.2022.890218

**Published:** 2022-07-26

**Authors:** Le-Xi Ding, Jing Zhang, Si-Si Yang, Jin Wu, Tong Su, Wei-Ming Wang

**Affiliations:** ^1^ Eye Center of Xiangya Hospital, Central South University, Changsha, China; ^2^ National Clinical Research Center for Geriatric Disorders, Xiangya Hospital, Changsha, China; ^3^ Hunan Key Laboratory of Ophthalmology, Central South University, Changsha, China; ^4^ Department of Oral and Maxillofacial Surgery, Center of Stomatology, Xiangya Hospital, Central South University, Changsha, China; ^5^ Institute of Oral Precancerous Lesions, Central South University, Changsha, China; ^6^ Research Center of Oral and Maxillofacial Tumor, Xiangya Hospital, Central South University, Changsha, China

**Keywords:** invadopodia-associated proteins, Hif1α, heat shock proteins 70, oral squamous cell carcinoma, bioinformatics analysis

## Abstract

**Background:**

Many studies have shown that diabetes is often closely related to oral squamous cell carcinoma (OSCC) occurrence and metastasis. Heat shock protein 70 (Hsp70) is a molecular chaperone related to diabetes complications. This study aims to investigate the role of Hsp70 in OSCC in expression of invadopodia-associated proteins.

**Methods:**

The expressions and correlation of HSP70, Hif1α, MMP2, MMP14, and cortactin were examined using bioinformatics analysis and verified by OSCC tissue microarrays. Assay *in vitro* was performed to analyze cell migration capacity after treatment with or without the HSP70 inhibitor.

**Results:**

The expressions of invadopodia-associated proteins were enhanced in OSCC tissues compared with paracarcinoma tissues and partially correlated with HSP70. Inhibiting HSP70 significantly decreased the cell viability, proliferation, and migration of OSCC cells.

**Conclusions:**

HSP70 may be involved in invadopodia-associated proteins in OSCC cells, which provides a promising method for treatment of OSCC metastasis.

## Introduction

Oral squamous cell carcinoma (OSCC) is diagnosed with 350,000–400,000 new cases every year around the world (1). Although the advanced multimodal treatment strategy has improved the prognosis and survival rate of OSCC patients, the 5-year survival rate still is about 50% these years (2, 3). The cause of occurrence and metastasis of OSCC is still unclear. Previous studies have proved that the occurrence and development of oral squamous cells may be closely related to diabetes (4).

The role of heat shock proteins 70 (Hsp70) in a variety of cancers and diabetes has been paid more attention in recent years. The expression level of HSP70 was demonstrated to be elevated remarkably in various cancers (5). The high expression level of Hsp70 is associated with the tumor resistance to chemotherapeutic agents (6, 7). HSP70 may play an important role in diabetes mellitus through a mechanism that interferes with the expression of cytokines, matrix metallopeptidase 9 (MMP9), and the antioxidant enzyme superoxide dismutase 2 (8–10). However, as far as we know, whether HSP70 is involved in the metastasis of oral cancer has not been reported.

Invadopodia, a subcellular membrane structure that can degrade the extracellular matrix (ECM), plays an essential role in the invasive migration and metastasis of cancer cells (11, 12). Proteinase like MMP2 and MMP9 are secreted at invadopodia to promote the degradation of ECM (13). The upregulation of some key invadopodia molecules in cancer cells including the matrix metalloproteinase MT1-MMP(MMP14) and the actin assembly protein (cortactin) is associated with patients poor prognosis (14). Tumor microenvironment such as growth factors, hypoxia, and PH was reported to affect the formation and function of invadopodia (15).

In this study, we attempt to explore the potential role of Hsp70 in OSCC expression of invadopodia-associated proteins. The cell viability, proliferation potential, and migratory ability of OSCC cells were examined after inhibiting Hsp70. We discovered that HSP70 may be important for the expression of invadopodia-associated protein.

## Materials and Methods

### Ethics Statement

The present study was approved by the Medical Ethics Committee of Xiangya Hospital, Central South University (Hunan, China) and was performed according to the Declaration of Helsinki guidelines on experimentation involving human subjects. Written informed consent was obtained from all participants.

### Patients

OSCC and matched paracancer tissues in the Department of Pathology, Department of Stomatology, Xiangya Hospital, Central South University were determined by two independent pathologists according to the 2006 WHO Classification System (16). In collaboration with Shanghai Biochip Co., LTD. (China), tumor tissue microarray was constructed, including 117 OSCCs and 56 matched paracancer tissues as previous described (17).

### Reagents, Antibodies, Cell Lines, and Culture

Apoptozole (APO, HSP70 inhibitor; catalog no. S8365) was purchased from Selleck China subsidiary (Shanghai, China). Antibodies HSP70 (catalog no. 4873), Hif1α (catalog no. 8690), cortactin (catalog no. 3502), and MMP2 (catalog no. 40994) were acquired from Cell Signaling Technology (Danvers, MA, USA). MT1-MMP (MMP14; catalog no. 14552-1-AP) was purchased from Proteintech (Wuhan, China). Human OSCC cell line (Cal27) was purchased from ATCC. Cal27 cells were cultured in Dulbecco’s minimum essential medium (Hyclone, Logan, UT, USA), containing10% fetal bovine serum (Gibco, Carlsbad, CA, USA) in oxygen concentrations for normoxia (21% O_2_) at 37°C in Anoxomat chambers (Mart Microbiology, Lichtenvoorde, the Netherlands).

### Immunohistochemistry

The sections of OSCC tissue arrays were deparaffinized in xylene and rehydrated in a graded series of ethanol and double-distilled water before subjected to heat-induced antigen retrieval. After incubated with primary antibodies (HSP70, 1:200; Hif1α, 1:100; MMP2, 1:200; MMP14, 1:50; cortactin, 1:100) overnight at 4°C, the secondary antibody was incubated at room temperature for 1 h. All slices were scanned by an Aperio ScanScope CS scanner (Epistem, Cambridge, MA) and quantified using Aperio Quantification software (Version 9.1, Epistem) for staining quantification. Histoscore of membrane and nuclear staining was calculated as a percentage of different positive cells using the formula (3+) ×3 + (2+) × 2 + (1+) × 1. Histoscore of pixel quantification was calculated as total intensity/total cell number. The threshold for scanning of different positive cells was set according to the standard controls provided by Aperio.

### Cell Counting Kit-8 Assay

When plated in 96-well plates at 2 × 10^3^ cells per well, Cal27 cells were cultured with Cell Counting Kit (CCK8; BioTime, Shanghai, China) for 24 and 48 h, respectively. Absorbance at 450nm was measured with the microplate reader (FlexStation 3, Molecular Devices, USA).

### EdU (5′-Ethynl-2’-Deoxyuridine) Staining Assay

The effects of Apo on cell proliferation were assessed by the Cell-Light™ 5′-ethynl-2′-deoxyuridine (EdU) Apollo^®^488 *In Vitro* Imaging Kit according to the manufacturer’s instructions. The number and proportion of the cells incorporated EdU were visualized and the fluorescence intensity was quantified using Image J1.42.

### Wound Healing Assay and Transwell Invasion Assay

Wound healing assay and Transwell invasion assay were performed as previously described (18) and details were described in Supplementary Material and Methods in S1 File.

### The Cancer Genome Atlas Analysis

In order to obtain a correlation between the target genes among head and neck cancer (HNSC) patients, gene expression quantification data of HNSC patients were downloaded from The Cancer Genome Atlas (TCGA) database. Then, the expression quantification data of HSPA4, HIF1A, MMP14, MMP2, and CCTN in TCGA HNSC dataset were extracted and processed by R × 64 3.5.1 with the package ggplot2, showing the inner relationship among above genes in HNSC intuitively. Search Tool for the Retrieval of Interacting Genes (STRING; https://string‐db.org/) was used to predict the interactions between proteins (19).

### Statistical Analysis

All experiments were performed at least in triplicate and each experiment was repeated for three times. GraphPad Prism Software (GraphPad Software Inc) was used for statistical analysis, and the data are presented as means ± standard error of mean (SEM). One-way analysis of variance (ANOVA) and Student’s t-test were used to analyze the differences as compared with control group and among each group. The relationship among the expression of Hsp70, Hif1α, MMP14, MMP2, and cortactin was examined using two-tailed Pearson’s statistics. The differences were considered statistically significant if the P-value < 0.05.

## Results

### The Overexpression of Hsp70, Hif1α, MMP2, MMP14, and Cortactin in OSCC

**Figure 1:**
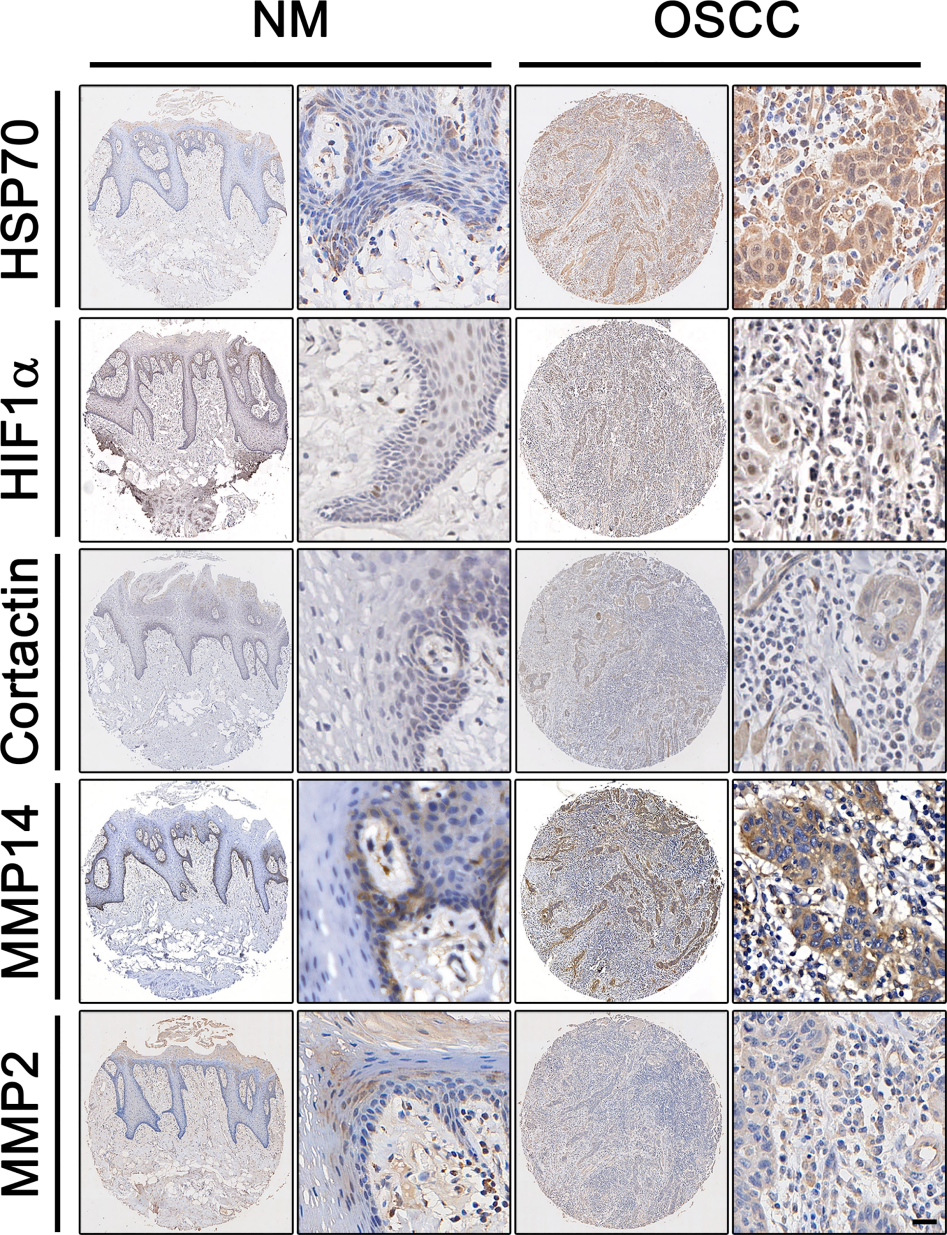
*Expression of HSP70, Hif1α, MMP2, MMP14, and cortactin in OSCC.* Representative immunohistochemical staining of HSP70, Hif1α, MMP2, MMP14, and cortactin expression in oral squamous cell carcinoma (OSCC) and matched paracarcinoma (NM). Scale bar, 50 μm.

**Figure 2:**
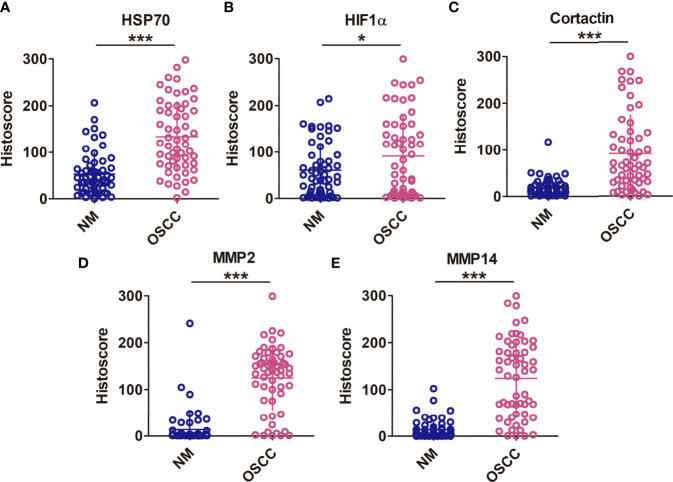
*High expression of HSP70, Hif1α, MMP2, MMP14, and cortactin in OSCC*. Histoscores of Hsp70 **(A)**, Hif1α **(B)**, cortactin **(C)**, MMP14 **(D)**, and MMP2 **(E)** in both tumor and normal tissues, and observed significantly higher expression levels of these proteins in OSCC tissues. Means ± SEM. *P < 0.05; ***P < 0.001 versus the NM group; Student’s t-test analysis.

We explored the expression of Hsp70, Hif1α, MMP2, MMP14, and cortactin in OSCC using tissue arrays, in which the normal adjacent tissues were used as control (denoted as normal mucosa). As shown in **Figure 1**, a significantly enhanced immunohistochemical staining was found in OSCC tissues compared with normal mucosa. The positive staining of Hsp70, MMP14, MMP2, and cortactin was mostly observed in the cytoplasmic of the carcinoma cells, whereas the nucleic positive signals were detected much more in the Hif1α immunohistochemical staining. The positive staining of MMP14 was mostly observed in the cytoplasmic and cytomembrane of the carcinoma cells. Then, we analyzed the histoscores of Hsp70 (**Figure 2A**), Hif1α (**Figure 2B**), cortactin (**Figure 2C**) MMP14 (**Figure 2D**), and MMP2 (**Figure 2E**) in both tumor and normal tissues and observed significantly higher expression levels of these proteins in OSCC tissues.

### The Correlations Among Hsp70, Hif1α, MMP2, MMP14, and Cortactin in OSCC

**Figure 3:**
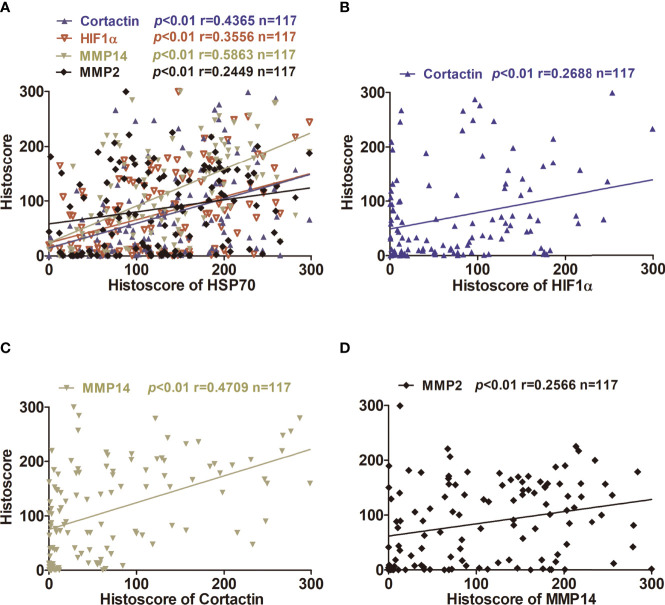
Correlation and regression of HSP70, Hif1α, MMP2, MMP14, and cortactin in OSCC tissues using two-tailed Pearson’s test. **(A)** A correlation and regression of HSP70, Hif1α, MMP2, MMP14, and cortactin in OSCC tissues using two-tailed Pearson’s test. **(B)** Correlation between Hif1α and cortactin expression levels in OSCC tissues. **(C)** Correlation between MMP14 and cortactin expression levels in OSCC tissue. **(D)** Correlation between MMP2 and MMP14 expression levels in OSCC tissues.

**Figure 4:**
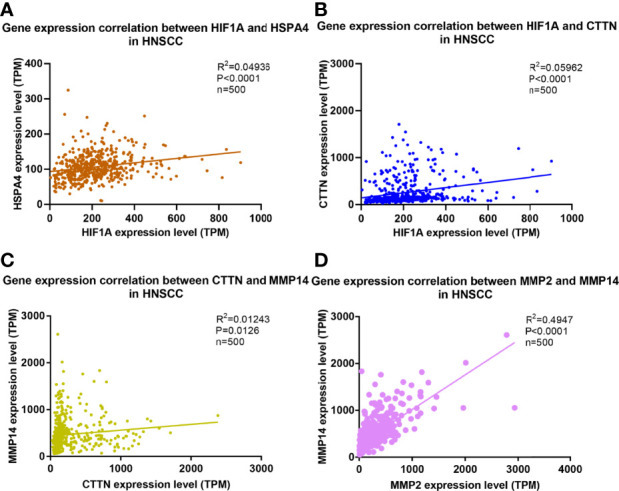
Correlation and regression of HSPA4, HIF-1A, MMP2, MMP14, and CTTN in TCGA database. Correlation and regression of **(A)** HSPA4, HIF-1A **(B)** HIF-1A, CTTN **(C)** CTTN, MMP14 **(D)** MMP2, and MMP14 in TCGA database.

In order to investigate the correlations among the expressions of Hif1α, MMP2, MMP14, cortactin, and Hsp70, we calculated the histoscores of Hsp70, Hif1α, MMP14, MMP2, and cortactin, and then picked every two proteins to analyze their correlations. Our results showed significant positive correlations between the histoscores of Hif1α, cortactin, MMP2, MMP14, and Hsp70. Moreover, the expression of cortactin in OSCC was positively correlated with the expression of Hif1α and MMP14, and the histoscores of MMP14 and MMP2 also showed a positive relationship (**Figures 3A–D**). We also extracted the above genes in OSCC samples from TCGA database and analyzed their correlations. As shown in **Figure 4**, the expression of HSPA4 and HIF1A (**Figure 4A**), HIF1A and CTTN (**Figure 4B**), CTTN and MMP4 (**Figure 4C**), as well as MMP14 and MMP2 (**Figure 4D**) also exhibited a significant positive relationship.

### Inhibiting Hsp70 Suppresses Cell Viability and Proliferation in OSCC Cells

**Figure 5:**
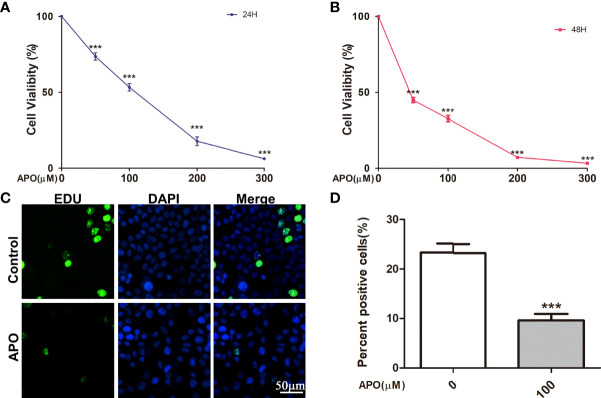
APO inhibits OSCC cell proliferation *in vitro.* Cell Counting Kit (CCK8) assay shows the suppressive effect of APO after 24-h **(A)** and 48-h **(B)** treatment by CCK8 assay and 5′-ethynl-2′-deoxyuridine (EdU) assay **(C)**. Means ± SEM; ^***^ *P* < 0.001; two-way ANOVA analysis. **(D)** Quantification of EdU assay. Means ± SEM; ^***^ *P* < 0.001; Student’s t-test analysis; APO, apoptozole.

In this study, Cal27 cells were treated with ascending concentrations of Apo for 24 h, and it was displayed in **Figure 5A** that the OSCC cell viability was reduced dose-dependently as the Apo concentration increased. The suppressive effect of inhibiting Hsp70 on OSCC cells viability was more evident after 48-h treatment (**Figure 5B**). Furthermore, we examined the possible role of inhibiting Hsp70 in cell proliferation using EdU incorporation assays. Compared with control group, Apo treatment group showed decreased EDU positive stained cells significantly (**Figure 5C**). The quantitative data suggested that, at 100 μM, Apo suppressed more than 50% proliferative potentials of OSCC cells (**Figure 5D**). In summary, inhibiting Hsp70 significantly reduced cell viability and inhibited cell proliferation in OSCC cells, exhibiting OSCC cell cytotoxicity.

### Inhibiting Hsp70 Prevents Cell Invasion of OSCC Cells

**Figure 6:**
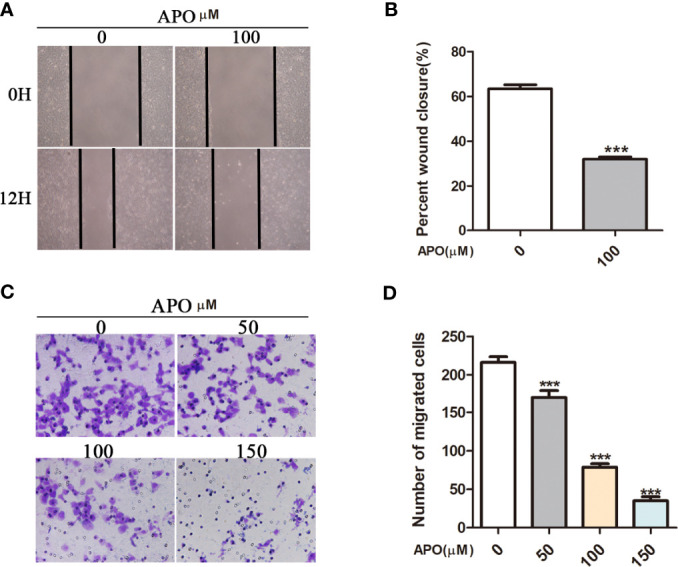
APO inhibits OSCC cell migration and invasion *in vitro.* Migration assessed by *in vitro* wound-healing assay **(A, B)** quantification of EdU assay. Means ± SEM; ^***^ *P* < 0.001; Student t-test analysis. Invasion assessed and by *in vitro* Transwell assay (8-μm pore size) **(C, D)** quantification of EdU assay. Means ± SEM; ^***^ *P* < 0.001; two-way ANOVA analysis; APO, apoptozole.

To further investigate the potential role of inhibiting Hsp70 in tumor invasion and metastasis, we employed wound healing assays and utilized Transwell chamber system to determine the effect of inhibiting Hsp70 on cell invasion. As **Figure 6A** showed, after treating 100 μM Apo for 12 h, Cal27 cells migrated more slowly in contrast to those treated without Apo. The motility of Cal27 cells was significantly inhibited after Apo treatment (**Figure 6B**). To further explore the correlation between ability of OSCC cells invasion and Apo concentration, Transwell chamber system was utilized in Cal27 cells at 0, 50, 100, and 150 μM Apo, respectively. It is revealed that the number of migrated cells significantly decreased as the Apo concentration improving in OSCC cells (**Figures 6C, D**).

## Discussion

In previous studies, HSP70, overexpressed in a variety of cancers, can inhibit endogenous and exogenous apoptotic pathways, block oncogene-induced senescence, and lead to treatment resistance (20). HSP70 also mediates the occurrence of tumor-promoting immune microenvironment through TLR4, NF-κB, STAT3, and other signal pathways (20, 21). The HSP70-TLR2 interaction also leads to the activation of neutrophils and the production of pro-inflammatory cytokines in diabetes vascular complications (20). Silencing of Hsp70 enhanced the metastatic properties of the HeLa, A549, and MCF7 cancer cell lines, and Hsp70 (HSP70A1A) inhibited the metastatic ability of cancer cells (22). However, heat shock protein family members such as HSC70 and HSPA2 may play inhibitory roles in cancer cell invasion and metastasis (23, 24). In OSCC, the relationship between HSP70 and tumor metastasis is unclear.

In our study, the Hsp70, Hif1α, cortactin, MMP2, and MMP14 expressions were elevated in OSCC tissue compared with normal counterparts. Cortactin is widely recognized as a marker of invadopodia. The main function of cortactin is to facilitate the interaction of Arp2/3 with F-actin to induce the formation of branched actin thereby promoting cell migration and invasion (25). Moreover, cortactin participates in matrix degradation and MMP2, MMP9, and MT1-MMP secretion in OSCC cells (26). Cortactin phosphorylation being regulated through Src-family kinases, Erk1/Erk2, and PAK is essential for invadopodia formation and ECM degradation (27). The recruitment and activation of proteases such as MMP2 and MMP9 at invadopodia sites could promote the degradation of ECM so as to provide traction for cancer invasion and metastasis (28, 29). In addition, MT1-MMP also contributes to the activation of MMP2 in invadopodia and facilitate the digestion of various ECM macromolecules including collagen, fibronectin, and laminins (30).

To evaluate the relationship between the expression of invadopodia-associated proteins and Hsp70, we examined histoscore of those proteins using two-tailed Pearson’s statistics. The results showed that positive relationships between Hsp70 and Hif1α, cortactin, MMP2, and MMP14 were found in OSCC tissue. Thus, we speculated that Hsp70 overexpression may be correlated with the increased invadopodia formation in OSCC. Bioinformatics on HNSC extracted from TCGA database also showed these positive relationships at the gene transcription level.

The life span of Hif1α could be prolonged owing to the formation of persistent complex between Hsp70 and Hif1α (31). HSP70 maintains Hif1α stability dependent on the activation of phosphatidylinositol 3-kinase (PI3K)/Akt and PI3K inhibitors could downregulate HSP70 expression (32). Hif1α not only increases the levels of invadopodia-forming activity but also stimulates its increased degradative activity (15, 33). Diaz et al. found that hypoxia no longer increases invadopodia formation after Hif1α knockdown in HNSC SCC61 cell lines, and it was believed that stabilized Hif1α under hypoxia activated Notch signaling thus increasing invadopodia formation (34). Moreover, Hif1α was reported to elevate the expression of b-PIX which was identified as a fundamental driver of invadopodia formation so that augments the invasive potential in cancer cells (33). Through a literature review, we prospected that inhibiting Hsp70 may affect the formation of invadopodia through downregulating the expression of Hif1α.

In order to further explore the role of Hsp70 in OSCC cells, we cultured Cal27 with distinct concentrations of Hsp70 inhibitor, Apo. We found that Apo could not only impair the cell viability and proliferation ability of OSCC cells in a dose-dependent manner but also attenuate their migratory ability. Overall, the *in vitro* experiment suggested that inhibiting Hsp70 may cause cytotoxicity to OSCC cells and interrupt their motility. These results further confirmed that HSP70 was involved in regulating the migration ability of OSCC cells.

It is worth mentioning that this study has some limitations. First, experiments on animals actually are needed to further verify the effect of Apo on OSCC. Last, our study discussed a little about the signaling mechanism of APO inhibiting the expression of Hif1α in OSCC cells, which may be meaningful to explore in the future.

## Conclusion

In conclusion, our work shows that HSP70 inhibition exhibits cytotoxic effect on OSCC cells and prevents invasion and metastasis of OSCC cells through decreasing the expression of invadopodia proteins. These results may provide a new therapeutic prospect in OSCC treatment.

## Data Availability Statement

The original contributions presented in the study are included in the article/**Supplementary Material**. Further inquiries can be directed to the corresponding author.

## Ethics Statement

The studies involving human participants were reviewed and approved by the Medical Ethics Committee of Xiangya Hospital, Central South University (Hunan, China). The patients/participants provided their written informed consent to participate in this study.

## Author Contributions

L-XD: concept/design, data analysis. W-MW: concept/design. S-SY, JW, JZ: data analysis. TS, W-MW: critical revision, final approval. All authors contributed to the article and approved the submitted version.

## Funding

This study was funded by the National Natural Science Foundation of China (grant no.81702708,81873717,82170973,82070966), Natural Science Foundation of Hunan (grant no. 2018JJ3862), the Science and Technology Innovation Program of Hunan Province (grant no.2021RC3026), Scientific research Project of Hunan Provincial Health Commission(grant no. 202208024658).

## Conflict of Interest

The authors declare that the research was conducted in the absence of any commercial or financial relationships that could be construed as a potential conflict of interest.

## Publisher’s Note

All claims expressed in this article are solely those of the authors and do not necessarily represent those of their affiliated organizations, or those of the publisher, the editors and the reviewers. Any product that may be evaluated in this article, or claim that may be made by its manufacturer, is not guaranteed or endorsed by the publisher.
