# Supplementary material for: Heat Shock Proteins 70 Regulate Cell Motility and Invadopodia-Associated Proteins Expression in Oral Squamous Cell Carcinoma
Source: Front Endocrinol (Lausanne). 2022 Jul 26;13:890218. doi: 10.3389/fendo.2022.890218 (PMC9362981; doi:10.3389/fendo.2022.890218)
Supplement: Supplementary file 1 [file DataSheet_1.pdf]

Supplementary Materials and methods

1.3 Wound healing assay

Cal27 cells ($5x10^5$) were placed and culture in 6-well plates overnight. A sterile 10-µl pipette tip was used to create wounds in 6-well plates. The cells were washed with PBS and cultured with fresh medium with or without Apo. After incubation for further 12h, an inverted microscope was used to determine the wounds.

1.4 Transwell invasion assay.

Transwell migration assay was conducted in a Transwell chambers (Corning, New York, NY, USA) with a polycarbonate membrane (8-µm polyester membrane filter pores). Cells were starved for 24 h prior to the experiment, and then $1x10^5$ cells were seeded into the upper chamber with 100 µl serum-free medium and ECM. The bottom chamber contained 800 µl of medium containing 10% FbS to serve as a chemoattractant. Following incubation at 37℃ for 48 h, the cells adhering to the lower surface of the membrane were fixed, stained, and captured.
