# Supplementary material for: Heat Shock Proteins 70 Regulate Cell Motility and Invadopodia-Associated Proteins Expression in Oral Squamous Cell Carcinoma
Source: Front Endocrinol (Lausanne). 2022 Jul 26;13:890218. doi: 10.3389/fendo.2022.890218 (PMC9362981; doi:10.3389/fendo.2022.890218)
Supplement: Supplementary file 2 [file DataSheet_2.pdf]

HSP70

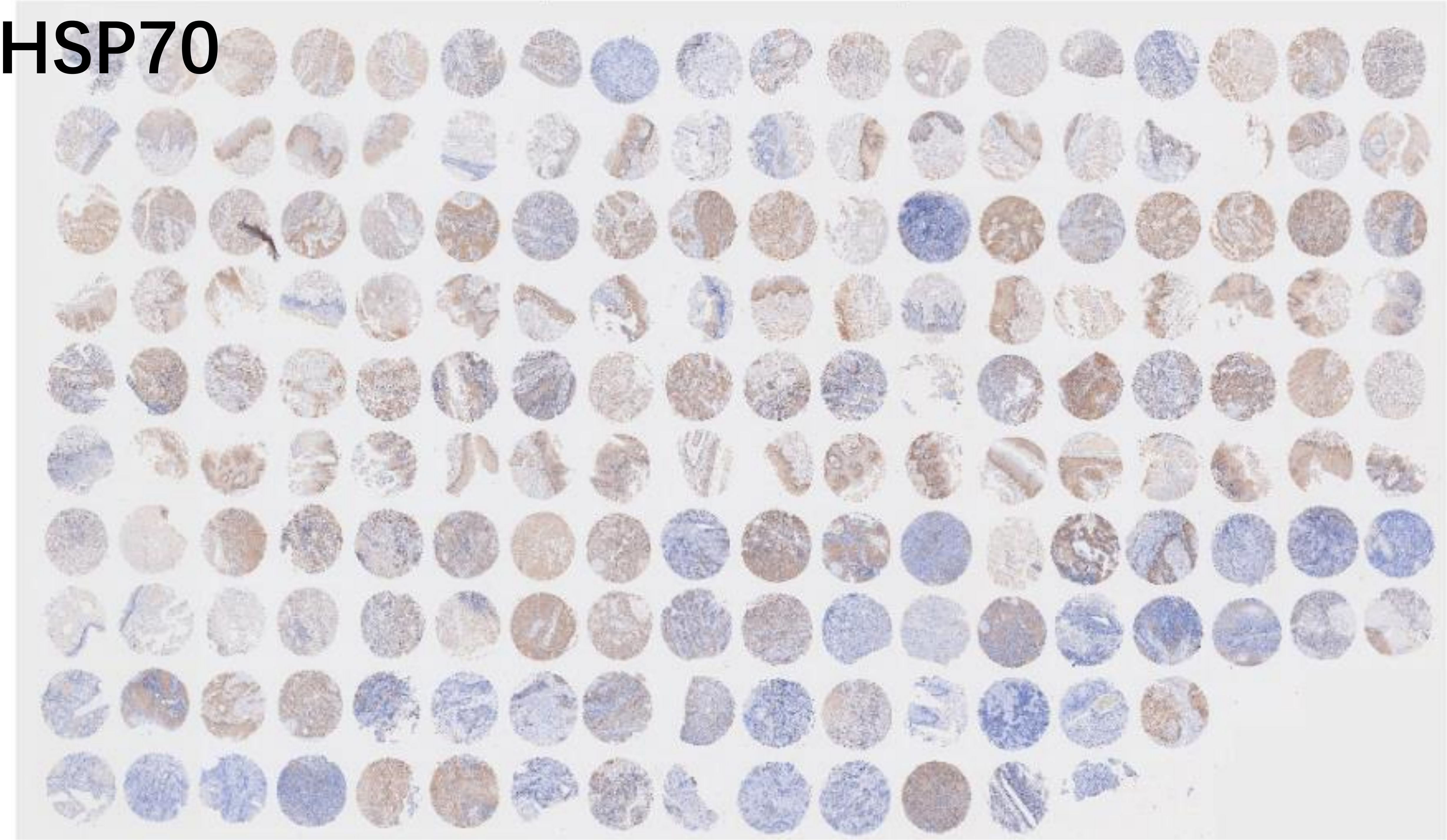

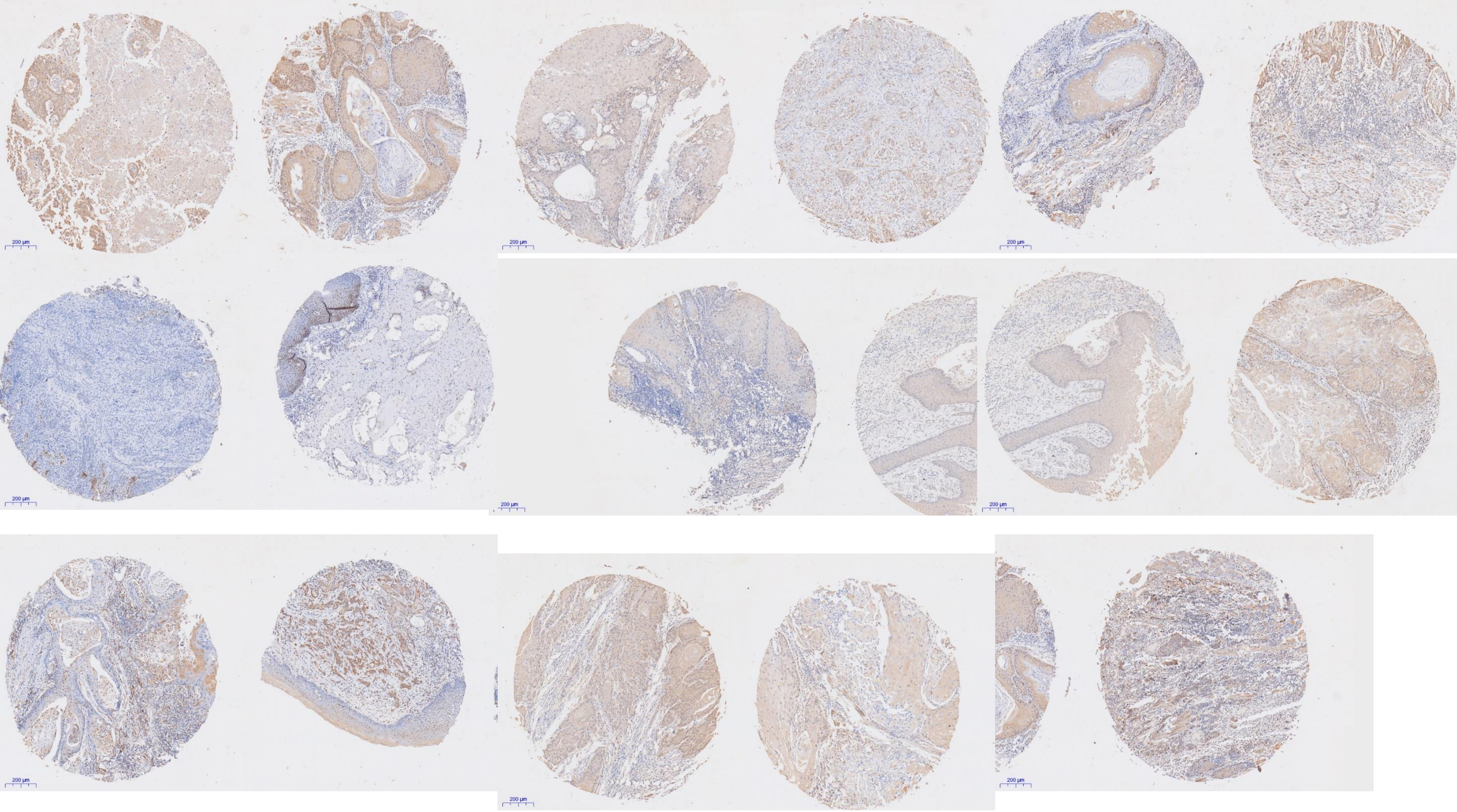
200 μm
200 μm
200 μm
200 μm
200 μm
200 μm
200 μm
200 μm
200 μm

HIF1α

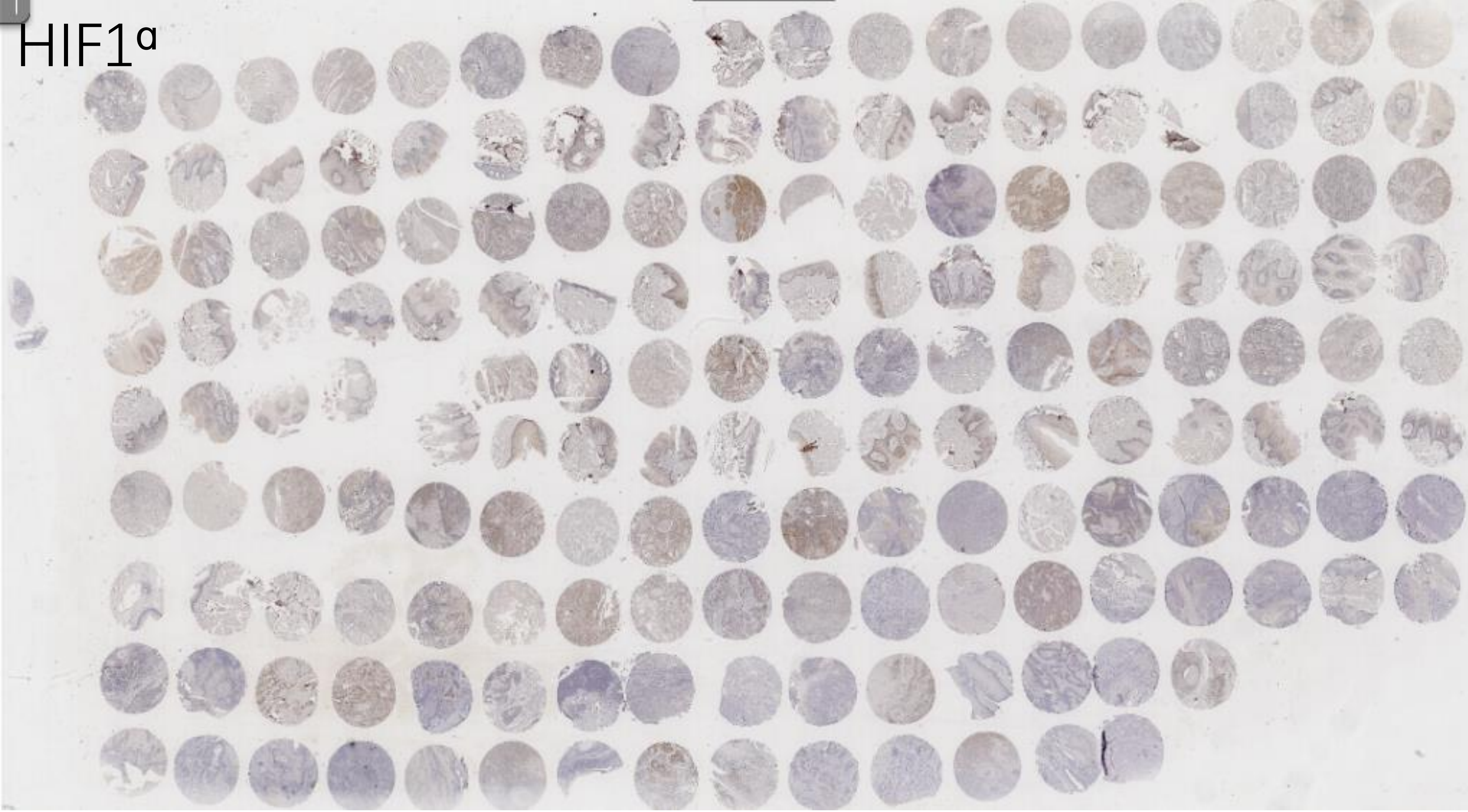

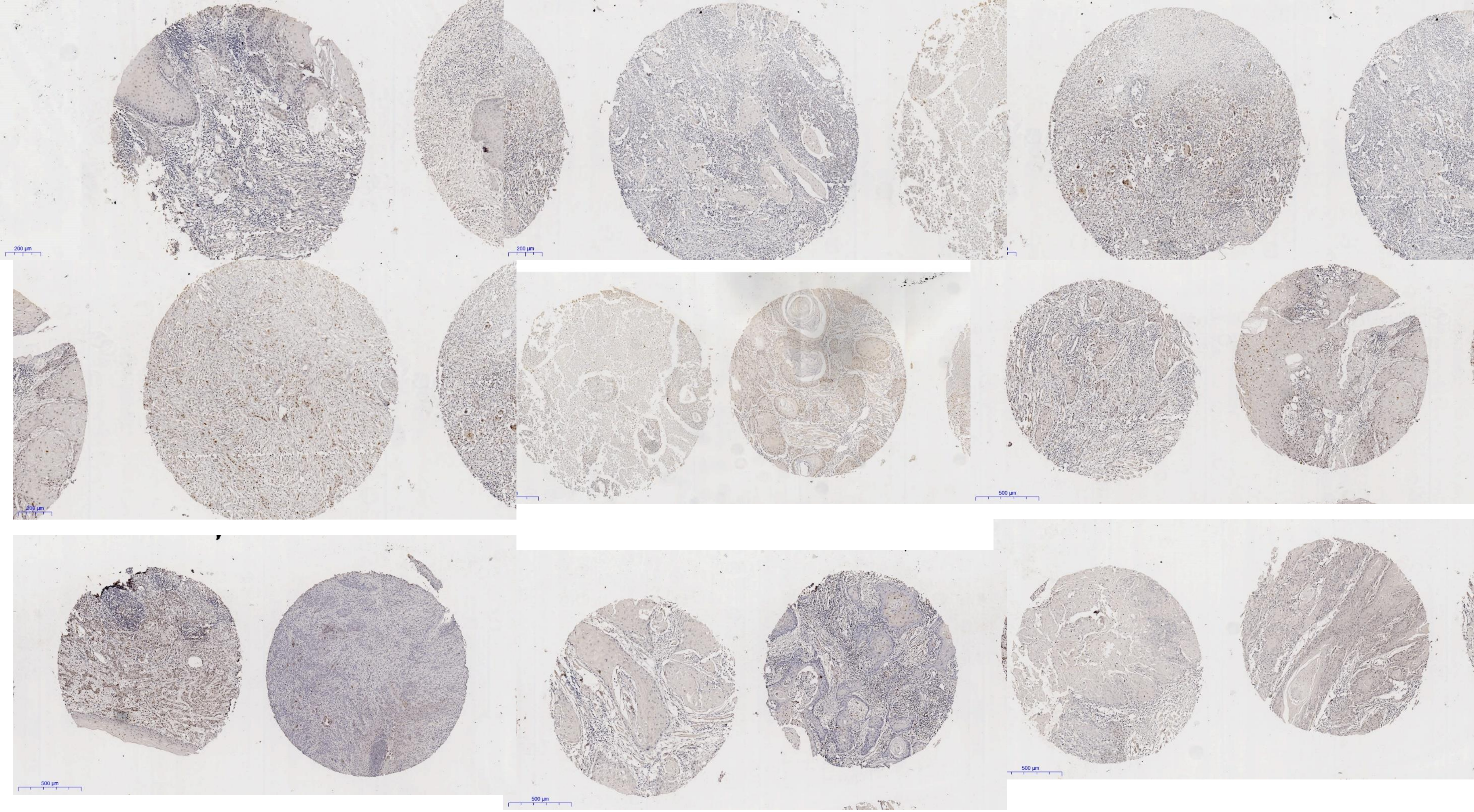
200 μm
200 μm
200 μm
500 μm
500 μm
500 μm
500 μm

CORTACTIN

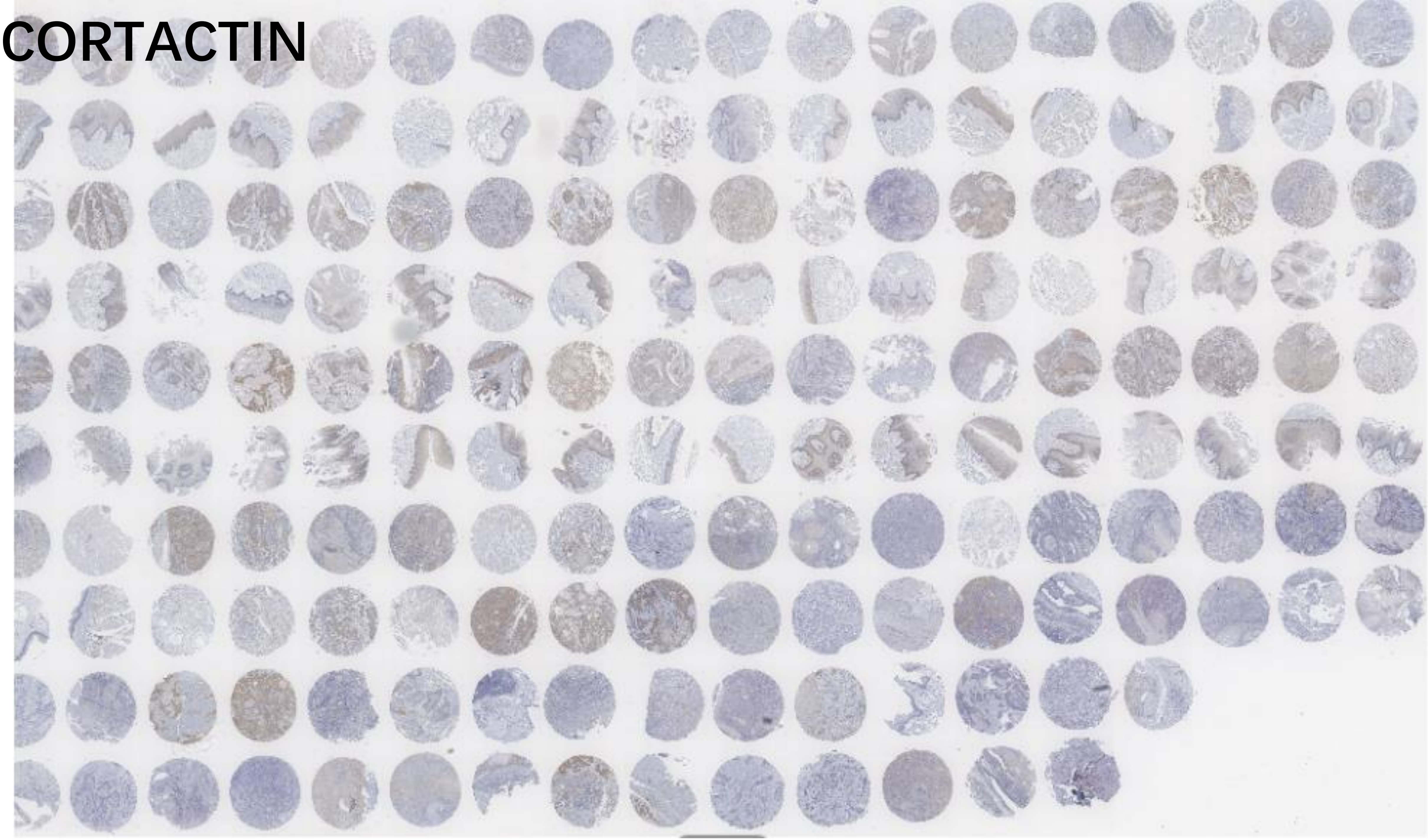

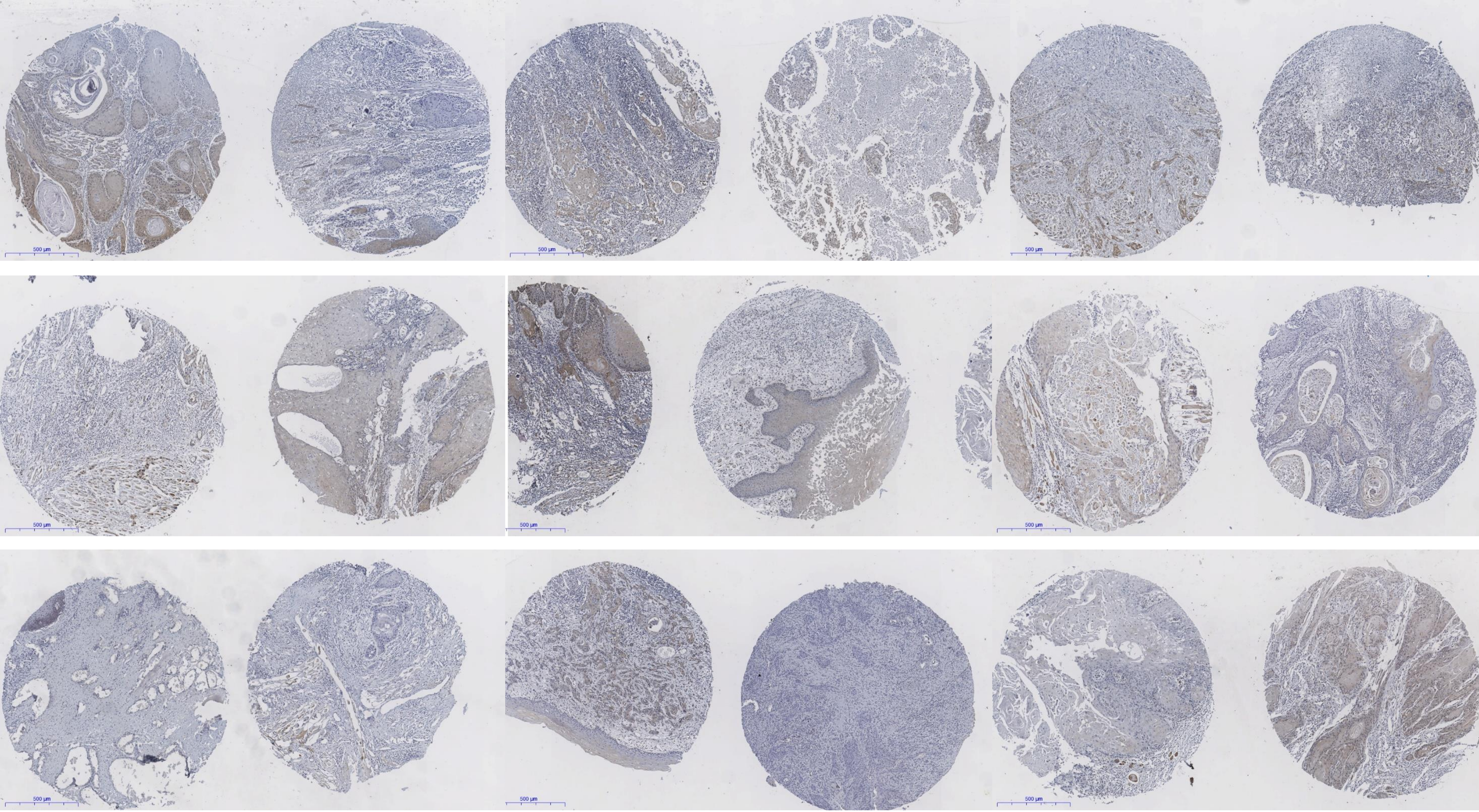
500 µm

MMP14

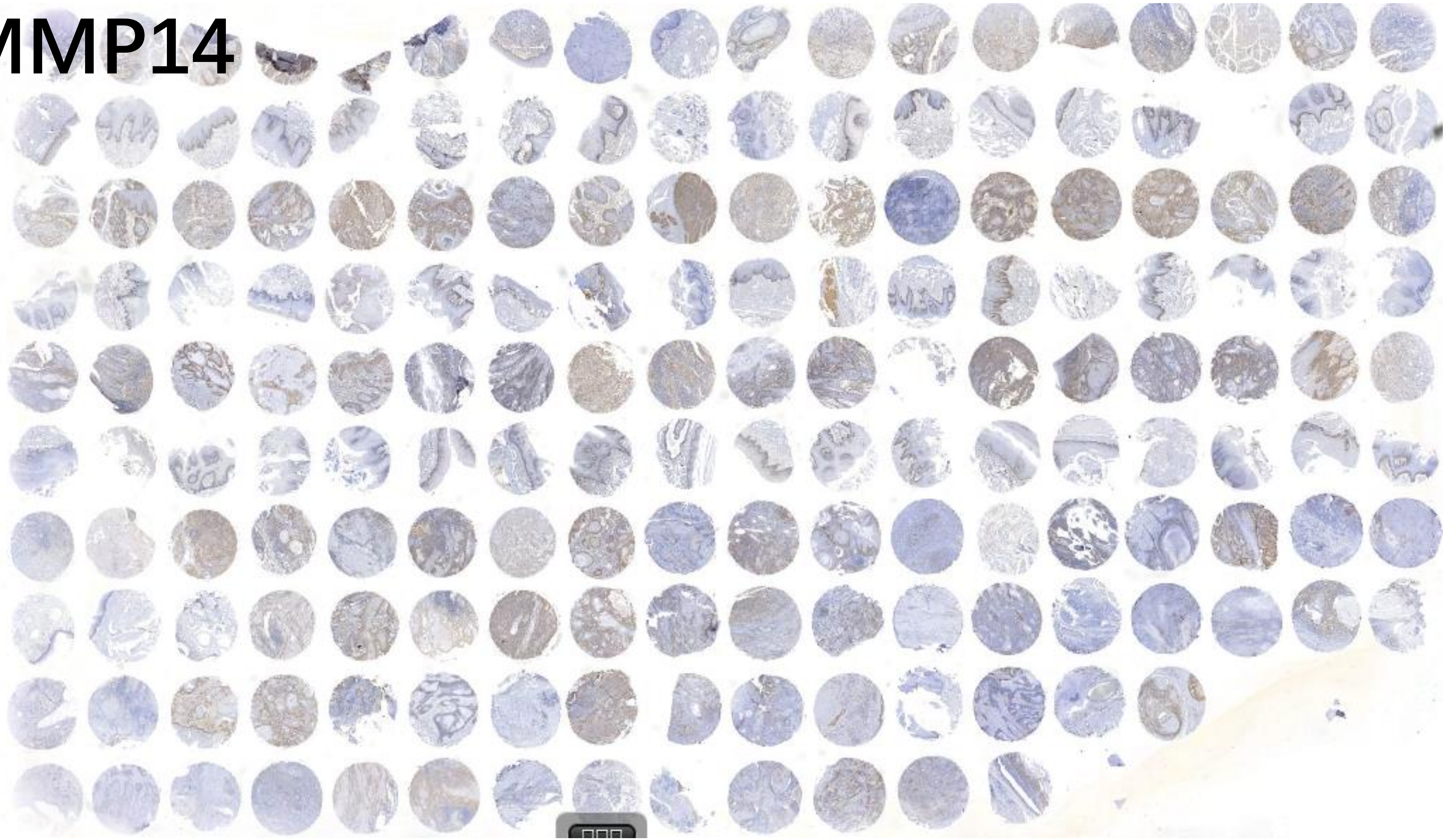

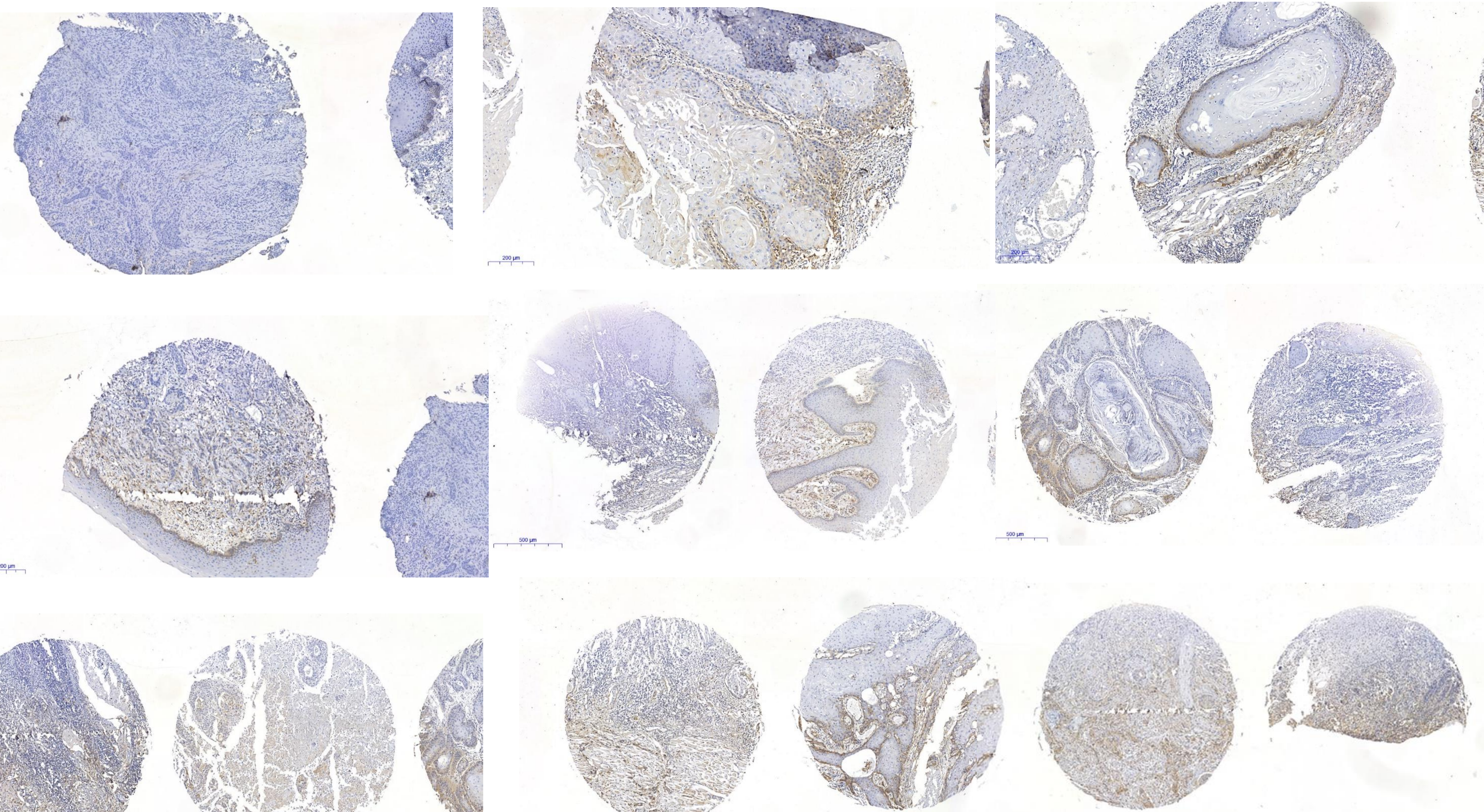
200 µm
200 µm
500 µm
500 µm

MMP2

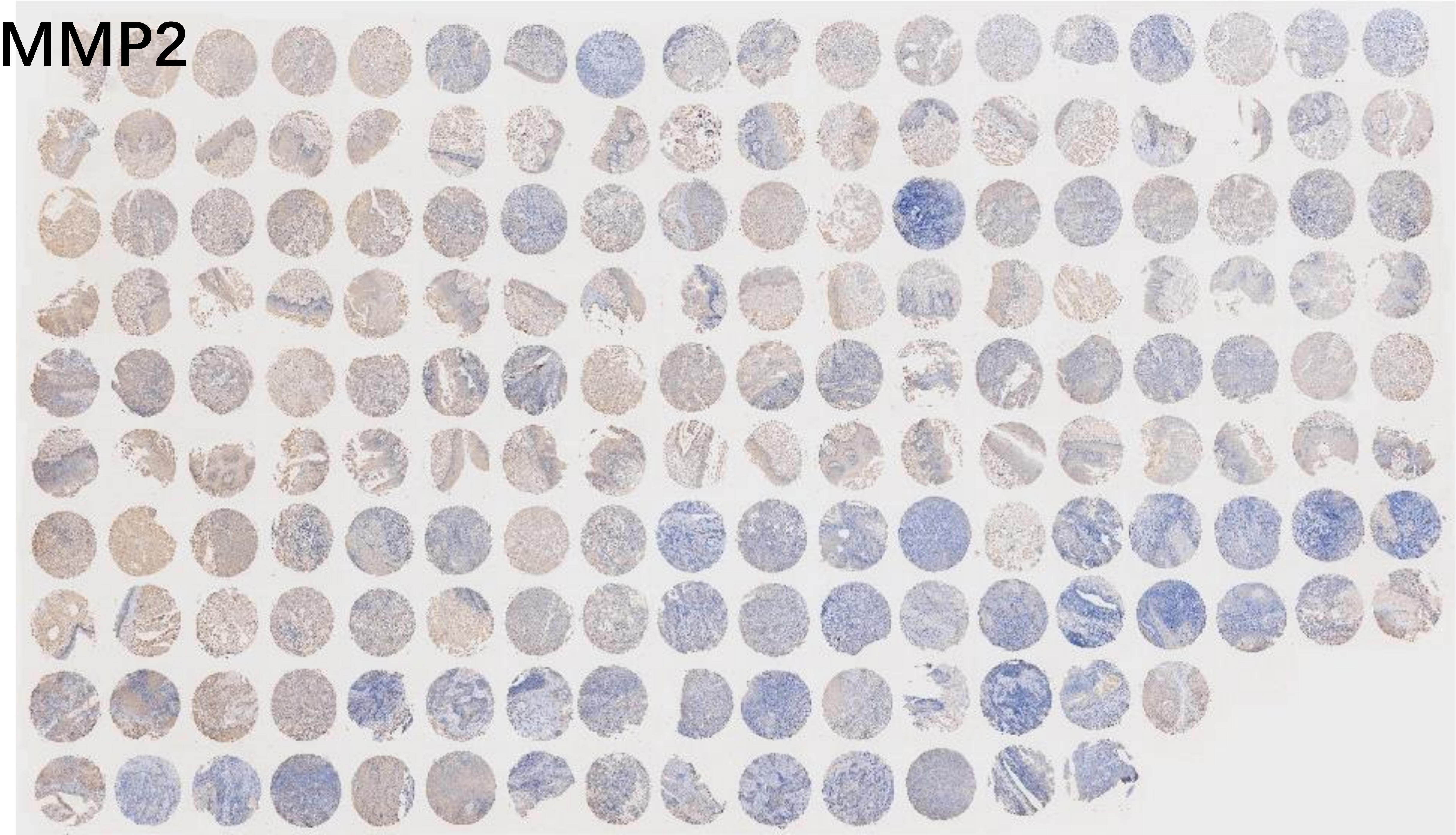

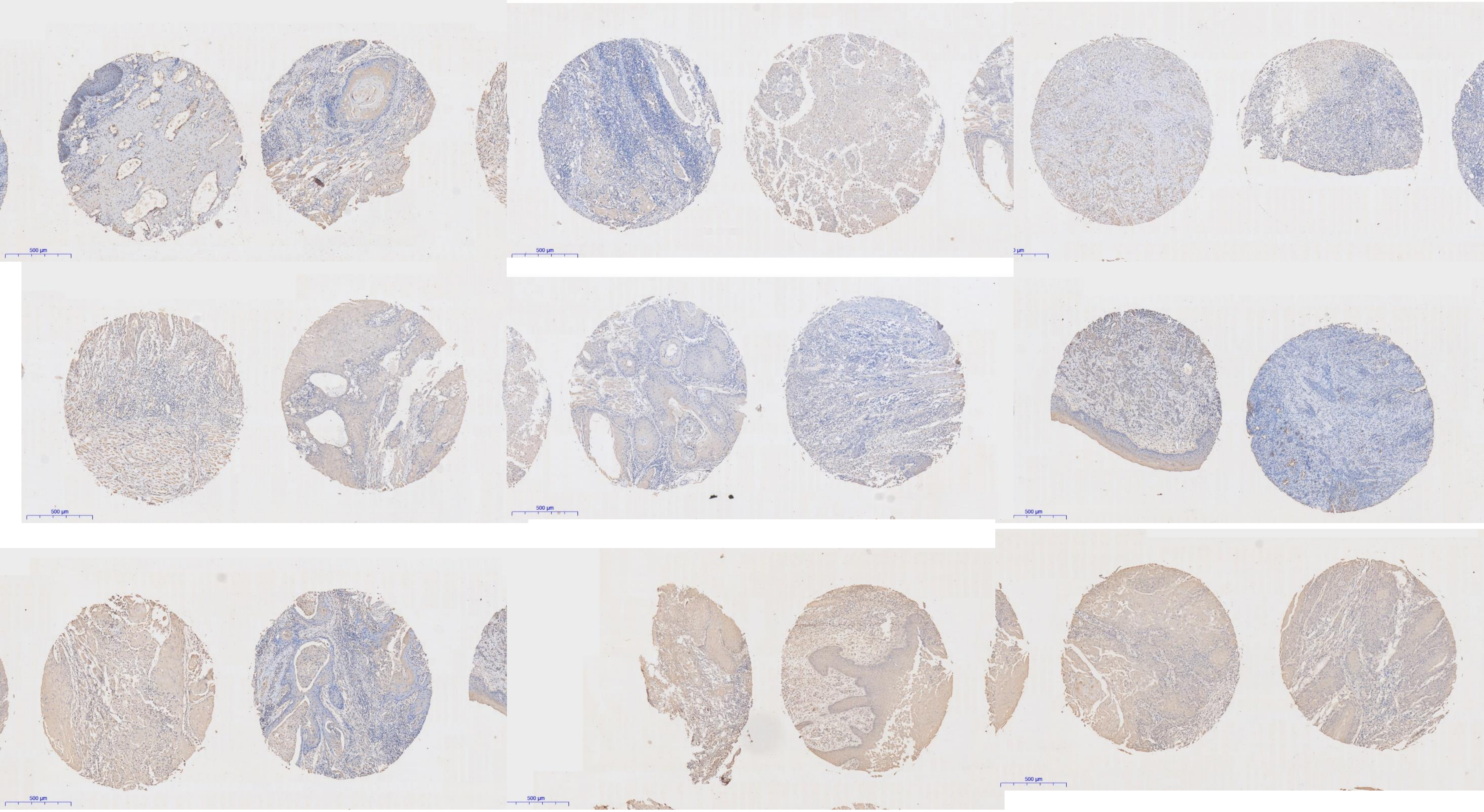
500 µm
500 µm
500 µm
500 µm
500 µm
500 µm
500 µm
500 µm

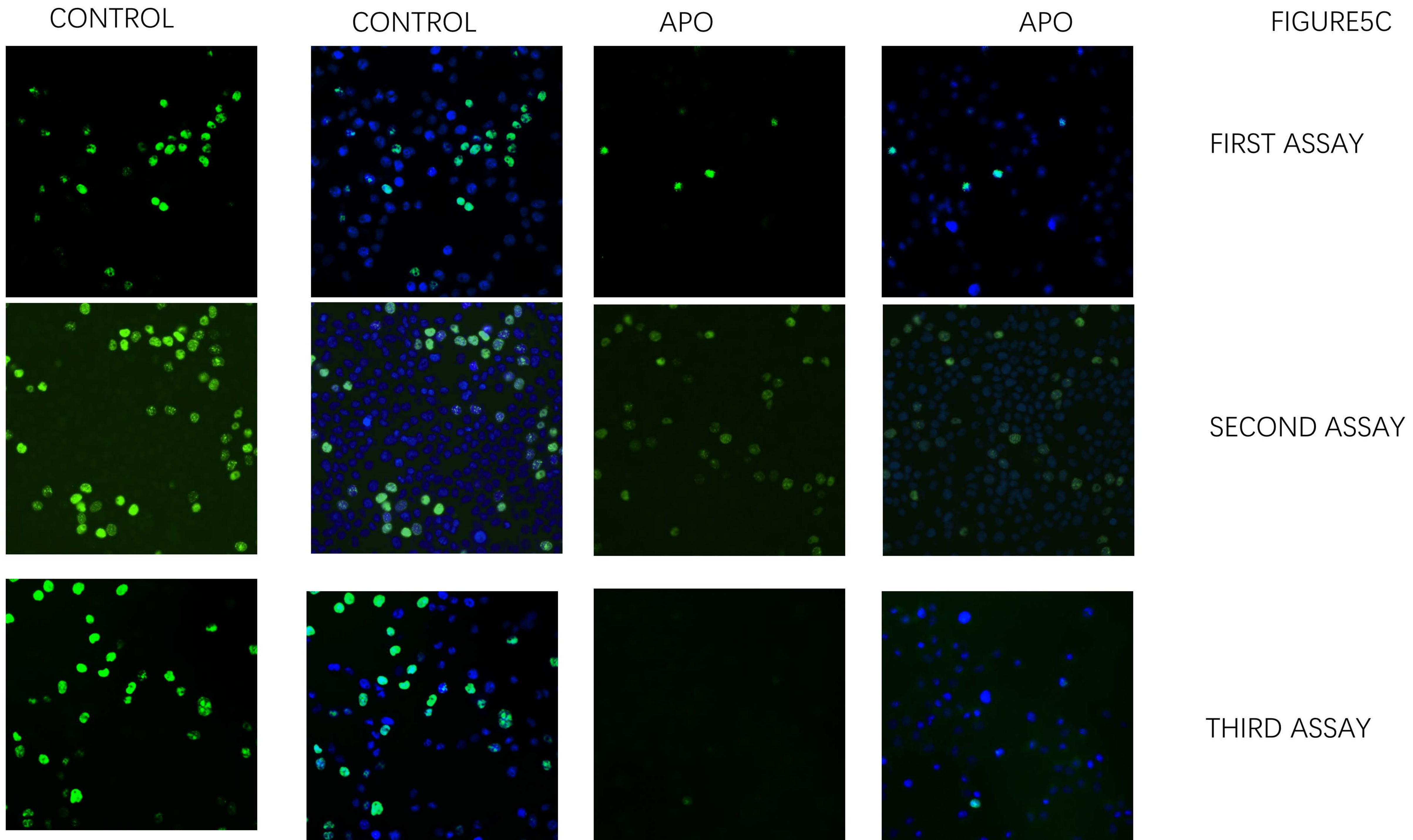
CONTROL
CONTROL
APO
APO
FIGURE5C
FIRST ASSAY
SECOND ASSAY
THIRD ASSAY

FIGURE6A

CONTROL

APO

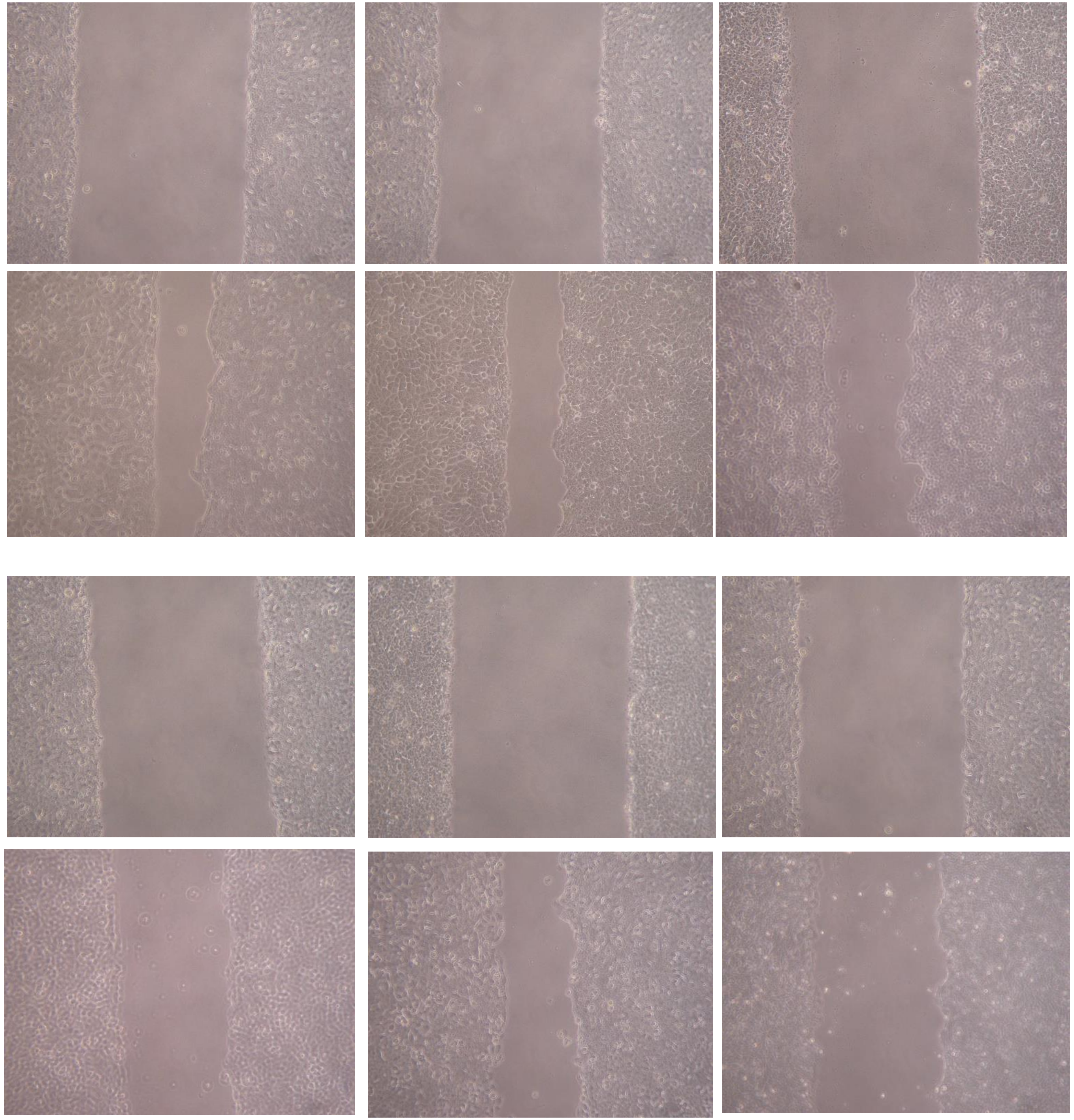

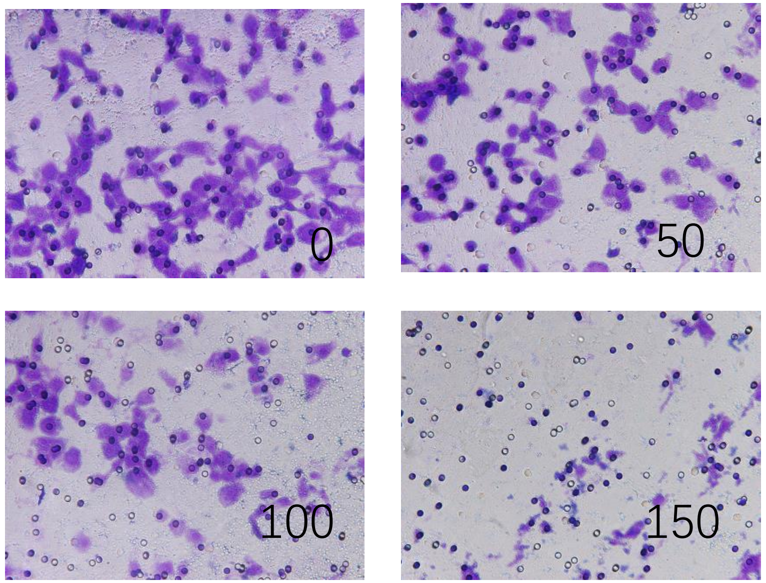

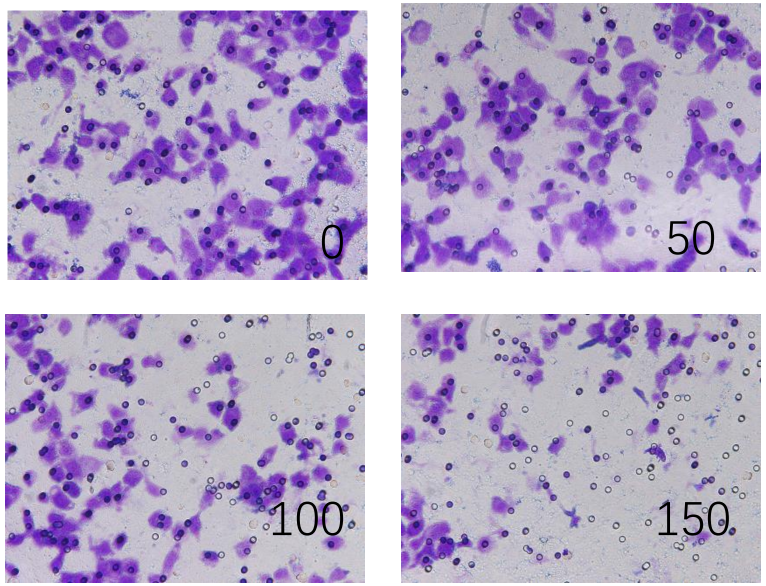

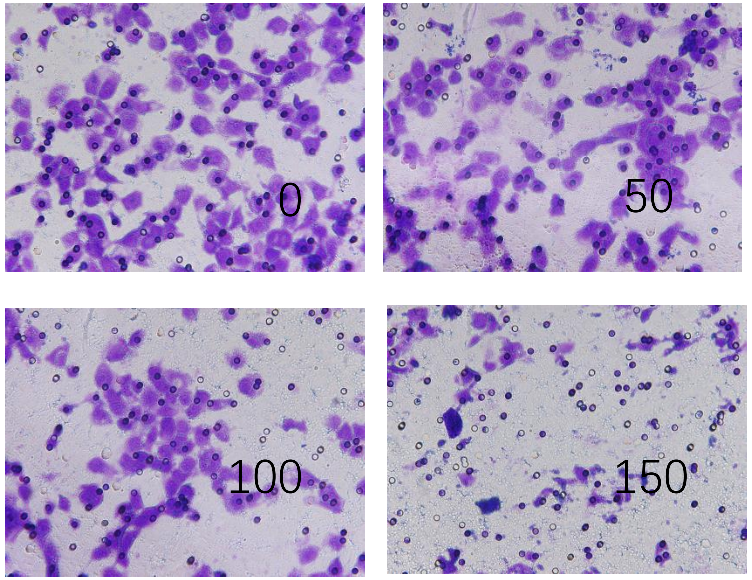

FIGURE6C
